# A procedure for estimating bias between quantitative analytical methods

**DOI:** 10.1155/S1463924686000287

**Published:** 1986

**Authors:** William C. Griffiths, Paul Camara, Israel Diamond, John C. Pezzullo

**Affiliations:** 1 Department of Laboratory Medicine Roger Williams General Hospital 825 Ckalkstone Avenue Providence Rhode Island 02908 USA; 2 Division of Biology and Medicine Brown University Providence Rhode Island USA; 3 Information Systems Department Rhode Island Hospital Providence Rhode Island USA


Journal of Automatic Chemistry/*Journal of Clinical Laboratory Automation*, Vol. 8, No. 3 (July–September 1986), pp. 147–150

### A procedure for estimating bias between quantitative analytical methods

**William C. Griffiths, Paul Camara, Israel Diamond**

*Department of Laboratory Medicine, Roger Williams General Hospital, 825 Chalkstone Avenue, Providence, Rhode Island 02908, USA, and Division of Biology and Medicine, Brown University, Providence, Rhode Island, USA*

**and John C. Pezzullo**

*Information Systems Department, Rhode Island Hospital, Providence, Rhode Island, USA*

### Introduction

Before quality control techniques and protocols can be applied to a laboratory procedure, the quality itself must be evaluated. This involves among other considerations an assessment of analytical accuracy. Some, but not all (and perhaps not even many), clinical laboratory analytical procedures can be considered *accurate* in the sense that every laboratory should get the same quantitative result for the analyte in question, regardless of the composition of the sample medium (for example potassium). That most methods are to one degree or another inaccurate does not invalidate their use. But the burden is on the laboratory, in choosing a method, to know just how accurate that method is.

If a new method can be shown to be sufficiently precise and stable over a period of weeks, and is linear and free of carry-over, then a few simple operations can assess and document its inaccuracy. One option is to determine the amount of bias that exists between the method under study (referred to as the *test* method) and a second *reference* method, the characteristics of which are presumably already known. An ample number of patient samples are analysed by both methods, preferably each of a pair being analysed on the same day. A plot of one method's results versus the other's is usually quite revealing—imprecision, non-linearity, and the presence of outliers are often evident from a visual inspection of the graph.

Problems arise, however, when more quantitative techniques are applied to the pairs of results. A simple least squares regression analysis is usually not valid here because it implicitly (and incorrectly) assumes that one of the two methods is essentially free of measurement error. The popular 'correlation coefficient' usually provides no added information—it is more appropriate for assessing departures from *no* correlation than for assessing departures from *perfect* correlation [1–3].

Instead of comparing the points to a *best-fitting* line it is generally more revealing to compare the points to the *line of identity* (the line passing through the origin with a slope of 1·0). A simple inspection of this plot will generally reveal, in addition to lack of linearity and the presence of 'outliers', any bias which may exist between the two methods. Obvious outliers (however defined) should be examined during the collection of data, preferably on or near the day of incidence. The sample in question should be reanalysed by both methods. A log should be kept of all gross random errors thus revealed. These may be instrument related or the result of human error. If the abnormal result persists on repeating the analyses, then an effort must be made to determine what characteristic of that sample matrix is causing the atypical behaviour.

Although plotting a set of 'test' method results versus those generated by a 'reference' method will probably show the presence of substantial bias between the methods, that procedure is relatively insensitive to *quantifying* the bias. A number of more rigorous alternatives to the construction of a simple least squares regression line have been suggested to accomplish this [1, 2 and 4]. With the exception of Lubran's technique, which employs multiple calculations of Student's t-test, these methods involve complex mathematics unfamiliar to most clinical scientists.

This paper presents a simple graphical technique for estimating the amount of bias between the results of two quantitative clinical methods, and for determining whether that bias is 'real' or just an artifact resulting from random sampling fluctuations. It is based on a statistical method developed by Bretaudiere and co-workers to study the suitability of control materials when used on different methods of assay of the same analyte [5].

### Method

The assay material for the comparison of methods should include human (patient) samples, as well as all control samples which are likely to be used in future monitoring of the method in question. As Bretaudiere points out, the data derived from this procedure may also be used to obtain information in reference to the suitability of a control sample for its designated purpose.

The material is analysed by both methods between which the bias is to be determined. The reference method may or may not be a true analytical reference method, but should be a method which is well studied and characterized as to its reproducibility and accuracy, including its response to interfering substances. In order to determine bias at different concentrations the patient samples should be grouped according to predetermined value ranges. This stratified analysis lets us distinguish *systematic bias* (in which the magnitude remains fairly constant over the entire range of test values) from

*proportional error* (in which the magnitude increases as the test value increases). Control material is also tested at different concentrations. Approximately 25 patient samples should be analysed by both methods, preferably at about the same time. Each control sample should be analysed approximately 10 times by both methods.

The pairs of results are then plotted, a line of identity is drawn, and the resulting graphs are examined visually for signs of non-linearity and bias. To determine whether the bias is real or merely due to random sampling fluctuations, given the imprecision of the two methods, the raw reference and test method data can be used to calculate a $z$-value [6]:

$$z = \frac{y - \mu_0}{s \div \sqrt{n}}$$

where: $y$ = mean of the raw *test* method results, $\mu_0$ = mean of the raw *reference* method results, $s$ = standard deviation of raw *test* method results, $n$ = number of pairs of results.

The $z$-value gives us a quantitative estimation of the significance of the observed bias. When $z > 1{\cdot}96$, the test method exhibits significant positive bias; when $z < -1{\cdot}96$, the test method exhibits significant negative bias.

Normalized test method data (the ratios of test method values to reference method values) could also be used in place of raw test method results in the formula above, with $\mu_0$ being set equal to 100. However, for results near zero, normalized values are subject to large fluctuations, even when only small differences between the two methods exist. In some circumstances, this might led to erroneous conclusions regarding intermethod bias.

When a number of related methods are being compared to their corresponding standard methods, the result can be displayed concisely by means of bar graphs of the normalized data, as illustrated in the following example.

### Example

Table 1 illustrates the raw and normalized data from a comparison of two sodium, two potassium, two chloride, and two carbon dioxide methods. The *reference* method for each was arbitrarily defined as that employed in the Beckman Astra-8 (Beckman Instruments, Brea, California, USA). The methods under examination (*test* methods) were sodium and potassium by flame photometry (IL 343, Instrumentation Laboratories, Lexington, Massachusetts, USA), chloride by coulometric-

*Table 1. Raw and normalized comparative data for Na, K, Cl, and $CO_2$. Mean, SD, SEM, and bias are indicated at the bottom.*

Electrolyte Data - Astra vs. Alternatives

| | <--- Sodium ---> | | | <-- Potassium --> | | | <-- Chloride --> | | | <----- CO2 -----> | | |
|---|---|---|---|---|---|---|---|---|---|---|---|---|
| Specimen | Astra | Flame | % | Astra | Flame | % | Astra | Coul. | % | Astra | Rate pH | % |
| #8 | 129 | 130 | 100.8 | 2.4 | 2.4 | 100.0 | 74 | 69 | 93.2 | 39 | 34 | 87.2 |
| #11 | 140 | 139 | 99.3 | 4.8 | 4.8 | 100.0 | 109 | 100 | 91.7 | 20 | 18 | 90.0 |
| #12 | 135 | 137 | 101.5 | 4.0 | 4.0 | 100.0 | 100 | 97 | 97.0 | 29 | 25 | 86.2 |
| #14 | 139 | 138 | 99.3 | 4.6 | 4.7 | 102.2 | 103 | 97 | 94.2 | 27 | 23 | 85.2 |
| #15 | 132 | 131 | 99.2 | 3.9 | 3.9 | 100.0 | 95 | 88 | 92.6 | 27 | 21 | 77.8 |
| #54 | 140 | 139 | 99.3 | 4.1 | 4.2 | 102.4 | 107 | 100 | 93.5 | 21 | 19 | 90.5 |
| #80 | 138 | 137 | 99.3 | 3.8 | 3.8 | 100.0 | 106 | 99 | 93.4 | 23 | 22 | 95.7 |
| #78 | 136 | 137 | 100.7 | 3.2 | 3.3 | 103.1 | 102 | 96 | 94.1 | 24 | 22 | 91.7 |
| #81 | 135 | 135 | 100.0 | 4.6 | 4.6 | 100.0 | 99 | 93 | 93.9 | 25 | 23 | 92.0 |
| #84 | 144 | 145 | 100.7 | 2.9 | 3.0 | 103.4 | 96 | 89 | 92.7 | 37 | 31 | 83.8 |
| #86 | 142 | 142 | 100.0 | 4.9 | 5.0 | 102.0 | 112 | 109 | 97.3 | 17 | 15 | 88.2 |
| #103 | 140 | 139 | 99.3 | 3.5 | 3.6 | 102.9 | 101 | 92 | 91.1 | 29 | 25 | 86.2 |
| #70 | 119 | 121 | 101.7 | 4.8 | 4.9 | 102.1 | 82 | 78 | 95.1 | 28 | 24 | 85.7 |
| #71 | 134 | 135 | 100.7 | 3.7 | 3.8 | 102.7 | 103 | 94 | 91.3 | 21 | 19 | 90.5 |
| #74 | 151 | 149 | 98.7 | 3.8 | 3.9 | 102.6 | 116 | 111 | 95.7 | 21 | 20 | 95.2 |
| #73 | 139 | 138 | 99.3 | 4.5 | 4.6 | 102.2 | 106 | 99 | 93.4 | 26 | 23 | 88.5 |
| #76 | 134 | 133 | 99.3 | 4.2 | 4.2 | 100.0 | 92 | 87 | 94.6 | 28 | 23 | 82.1 |
| #98 | 142 | 141 | 99.3 | 4.2 | 4.2 | 100.0 | 96 | 91 | 94.8 | 36 | 33 | 91.7 |
| #501 | 146 | 143 | 97.9 | 4.3 | 4.4 | 102.3 | 112 | 106 | 94.6 | 25 | 24 | 96.0 |
| #15/299 | 145 | 143 | 98.6 | 3.9 | 4.0 | 102.6 | 107 | 100 | 93.5 | 26 | 23 | 88.5 |
| #95 | 141 | 142 | 100.7 | 3.3 | 3.4 | 103.0 | 104 | 96 | 92.3 | 27 | 24 | 88.9 |
| Mean = | | | 99.8 | | | 101.6 | | | 93.8 | | | 88.6 |
| Std. Deviation = | | | 1.0 | | | 1.3 | | | 1.6 | | | 4.5 |
| Std. Error of Mean = | | | 0.2 | | | 0.3 | | | 0.4 | | | 1.0 |
| Average - 2 S. E. M. = | | | 99.4 | | | 101.0 | | | 93.1 | | | 86.7 |
| Average + 2 S. E. M. = | | | 100.2 | | | 102.2 | | | 94.5 | | | 90.6 |
| Biased? | | | No | | | Yes | | | Yes | | | Yes |

amperometric titration (Fiske Chloridometer, Fiske Associates, Uxbridge, Massachusetts, USA), and carbon dioxide by rate of pH change (Beckman Cl/$CO_2$ Analyzer, Beckman Instruments, Brea, California, USA). Twenty-one patient serum specimens were analysed by both methods for each analyte. The results of the test method were then plotted against the results of the corresponding reference method for the same analyte on linear graph paper (see figures 1, 2, 3 and 4). These graphs clearly show the upward bias in the potassium test method, the downward bias in the chloride test and carbon dioxide test methods, and the evident lack of bias in the sodium test method. On closer examination, one can further discern that the bias in the potassium and chloride methods is probably of the systematic type (the points appear to lie parallel to the line of identity), while the bias in the $CO_2$ method is more proportional (the points diverge farther from the line of identity for larger values of concentration).

The computations required to assess the significance of bias in the four methods are shown in table 1. The test method result for each sample was expressed as a percentage, relative to the reference method result for the same sample (normalized result). It is seen that in the case of sodium the average percentage does not differ from 100% by more than two SEMs, so there is no significant bias. The other three analyses are seen to have average percentages differing from 100% by more than two SEMs, indicative of significant bias in these methods.

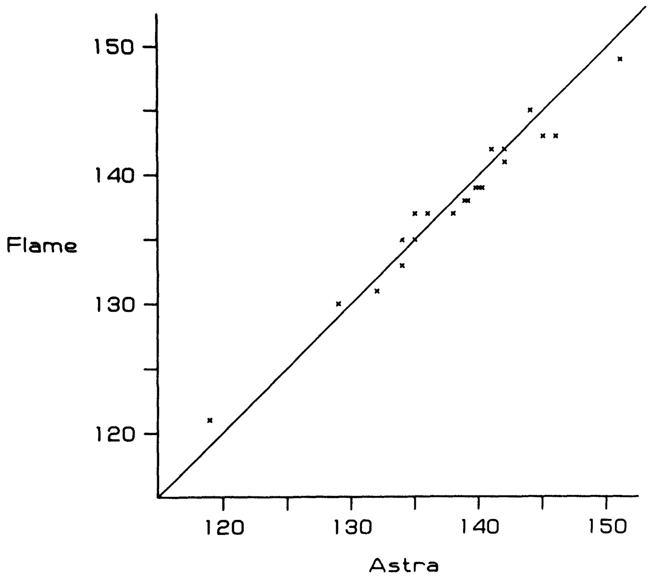

*Figure 1. Linear plot of Astra sodium results in mEq/l (x-axis) versus flame sodium results in mEq/l (y axis).*

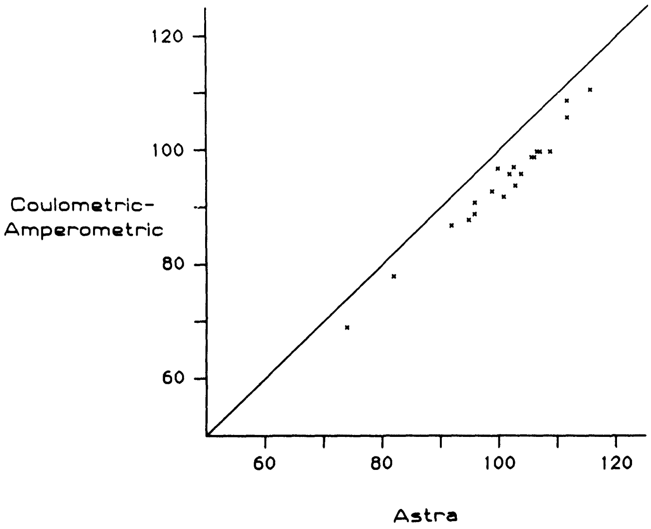

*Figure 3. Linear plot of Astra chloride results in mEq/l (x-axis) versus Fiske chloride results in mEq/l (y axis).*

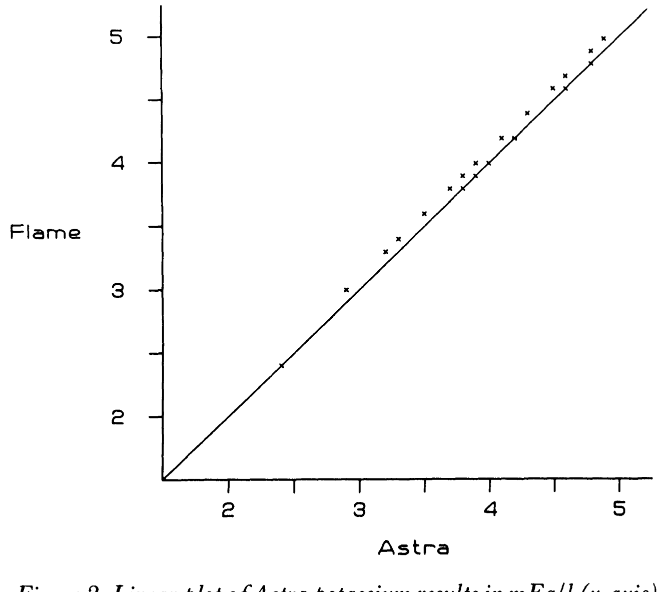

*Figure 2. Linear plot of Astra potassium results in mEq/l (x-axis) versus flame potassium results in mEq/l (y-axis).*

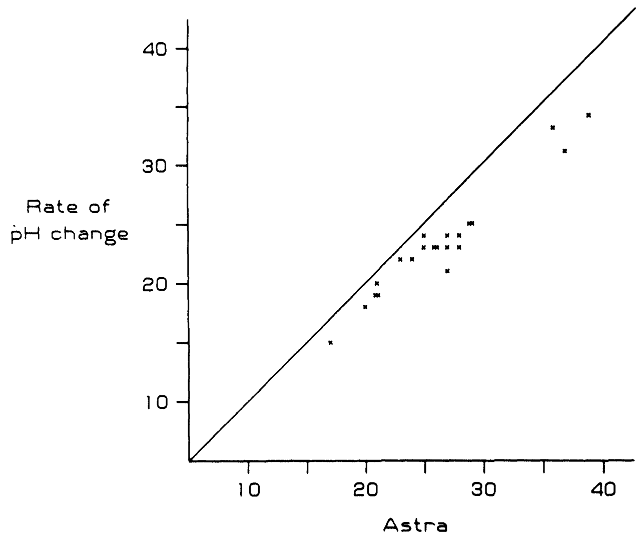

*Figure 4. Linear plot of Astra carbon dioxide results in mEq/l (x-axis) versus Beckman Cl/$CO_2$ Analyzer carbon dioxide results in mEq/1 (y-axis).*

The results of the significance testing are concisely expressed by the bar-graph in figure 5. In this graph, each of the four tests' bias is summarized by one bar figure, whose mid-point corresponds to the mean of the normalized results. The length of the line represents the mean ± one standard deviation, indicating the amount of scatter among the individual samples. If the values are normally distributed, the line spans a range which includes about 68% of the data points. The thick middle portion of the bar indicates the mean ± two standard errors of the mean (SEMs), indicating how precisely the average bias is known. If the thick bar crosses the 100% line, there is no significant evidence of bias; if it does not cross the 100% line, then significant bias is present.

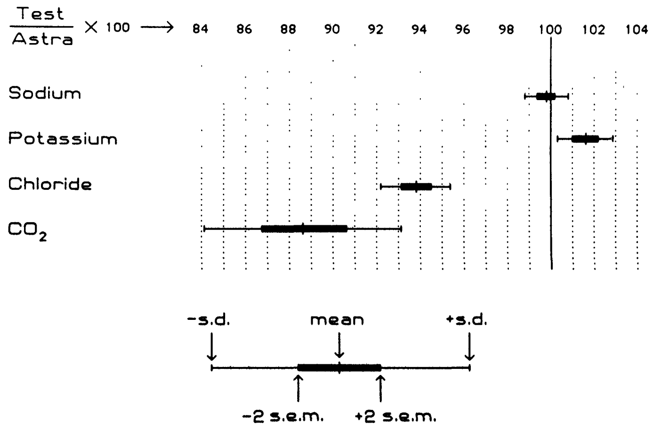

*Figure 5. Bar graph plot of normalized test method data indicating SD and SEM.*

A computer program is available which performs the linear plot of data from one method versus that of another, data normalization, and $z$-value calculation. It is available for the Apple II+/IIe with 48K RAM and one disk drive.

Even without a microcomputer, however, this method is a convenient, simple method of estimating bias.

Please note that the data in this paper does not constitute an evaluation of any of the methods used as examples. These methods are merely representative of the type for which this procedure for the estimation of bias is a useful statistical tool.

### Acknowledgements

The authors wish to thank Mrs Therese Souza for writing the computer program.

### Appendix

*Explanation of the $z$-value formula and critical value*

The error of a measured value of analyte (like a sodium concentration) can usually be thought of as arising from the combined action of a number of small, independent perturbations that occur at various stages in the analysis. The Central Limit Theorem leads us to expect that these measurement errors will be nearly normally distributed. When two values, each with a normally distributed error, are subtracted, the rules for error-propagation predict that the difference will also have a normally distributed error. If no bias were present between the two methods under study, we would expect the means of the reference and test methods' data to be equal. The question of intermethod bias then becomes a question of whether the means of the two sets of values differ significantly.

The appropriate statistical test is a Two Sample Student t-test, which we are simplifying by using only the test method values to estimate the standard deviation. The $z$-value formula given above is actually an approximation of the Student t-formula. This approximation is adequate for the use to which we are putting it. For large values of $n$, the Student t-distribution approaches the normal distribution, and considering the other assumptions and approximations used in the derivation, it is acceptable to approximate the critical Student t-value by the corresponding normal distribution critical value. For example, with sample sizes of 25 as recommended in this procedure, the critical value for the two-tailed Student t-test with $p = 0{\cdot}05$ is 2·06, not substantially different from the 1·96 value used in our procedure.

### References

1. Cornbleet, P. J. and Gochman, N., *Clinical Chemistry*, **25** (1979), 432.
2. Lubran, M. M., *Annals of Clinical and Laboratory Science*, **12** (1982), 134.
3. Wakkers, P. J. M., Hillendoarn, H. B. A., Op de Weegh, G. H. and Heerspink, W., *Clinica Chimica Acta*, **64** (1975), 173.
4. Parvin, C. A., *Clinical Chemistry*, **30** (1984), 751.
5. Bretaudiere, J. P., Dumont, G., Rej, R. and Bailly, M., *Clinical Chemistry*, **27** (1981), 798.
6. Baur, S. and Kennedy, J. W., *Journal of Clinical Laboratory Automation*, **3** (1983), 46.

